# Optimization of Spray-Drying Conditions and Storage Stability of Black Lychee Extracted Powder: Impact of Maillard Conjugation on Bioactive Compounds and Antioxidant Activity

**DOI:** 10.3390/foods15162883

**Published:** 2026-08-18

**Authors:** Supakit Chaipoot, Rewat Phongphisutthinant, Kuntathee Chaimueng, Sirinthip Jaijoi, Pairote Wiriyacharee, Chalermkwan Somjai, Worachai Wongwatcharayothin, Pattavara Pathomrungsiyounggul, Kanokwan Kulprachakarn

**Affiliations:** 1 Multidisciplinary Research Institute, Chiang Mai University, Chiang Mai 50200, Thailand; supakit.ch@cmu.ac.th (S.C.); rewat.p@cmu.ac.th (R.P.); kuntathee.c@gmail.com (K.C.); ninesrt1991@gmail.com (S.J.); 2 Center of Excellence in Microbial Diversity and Sustainable Utilization, Chiang Mai University, Chiang Mai 50200, Thailand; 3 Faculty of Agro-Industry, Chiang Mai University, Chiang Mai 50100, Thailand; pattavara.p@cmu.ac.th; 4 Processing and Product Development Factory, The Royal Project Foundation, Chiang Mai 50100, Thailand; pairote.w@cmu.ac.th (P.W.); chalermkwansomjai@hotmail.com (C.S.); 5 Faculty of Humanity, Chiang Mai University, Chiang Mai 50200, Thailand; worachai.w@cmu.ac.th; 6 School of Health Sciences Research, Research Institute for Health Sciences, Chiang Mai University, Chiang Mai 50200, Thailand

**Keywords:** functional food powder, Maillard conjugation, spray-drying, *Litchi chinensis*, antioxidant stability, maltodextrin encapsulation

## Abstract

The development of functional ingredients from perishable tropical fruits supports sustainable food systems by reducing postharvest losses while retaining bioactive compounds. This study developed and optimized black lychee (*Litchi chinensis* Sonn.) extracted powder (BLEP) by spray drying and evaluated its physicochemical characteristics and antioxidant stability during storage. A 2^2^ factorial design was employed to investigate the effects of maltodextrin concentration (5–30%) and inlet temperature (160–200 °C). Graphical optimization using an overlay plot identified 17.80% maltodextrin and an inlet temperature of 200 °C as the best compromise among the significant responses within the range tested. The optimized BLEP contained vanillic acid and epicatechin as the predominant phenolic compounds together with essential amino acids, including leucine. Degree of glycation (DG) and HMF were measured as indirect indicators of Maillard reaction progress. Over six months at 4, 25, 35, and 45 °C, color parameters, DG, and HMF remained stable, whereas total phenolic content (TPC) declined from 437.11 to 225–252 mg GAE/100 g db and ABTS radical scavenging activity declined progressively. TPC was strongly associated with antioxidant capacity (r = 0.914 with ABTS, *p* < 0.01), and its degradation followed zero-order kinetics (adjusted R^2^ = 0.931–0.971) with a predicted half-life (L_50_) of 199–220 days. The optimized powder therefore showed good physical and chromatic stability with partial loss of antioxidant-related compounds over time.

## 1. Introduction

Natural bioactive food ingredients are essential components in the formulation of innovative and nutritious food products, contributing to enhanced sensory quality, nutritional value, and functional properties [1,2]. The growing consumer demand for convenient, nutrient-rich food products has accelerated interest in fruit powders as value-added alternatives to perishable raw materials, particularly in nutraceutical and functional beverage applications [3]. Fruits are rich sources of bioactive compounds such as phenolics, flavonoids, and amino acids, which offer antioxidant benefits and health-promoting effects [4,5,6]. However, the perishability of many fruits necessitates advanced preservation techniques to extend shelf life while maintaining bioactive potential [7].

Lychee (*Litchi chinensis* Sonn.) is a phenolic-rich tropical fruit containing flavonoids (e.g., catechin, epicatechin, and rutin), phenolic acids, and essential amino acids that contribute to its antioxidant capacity and nutritional quality [8]. Our previous work reported that this thermal aging increased the concentrations of selected phenolic compounds and of several free amino acids, among which leucine was the most abundant [9]. Leucine is reported here as a compositional feature of the aged matrix [10]. In addition, high-temperature aging can promote complex Maillard reactions, influencing physicochemical characteristics and antioxidant potential. Temperature plays a critical role in modulating Maillard reactions, as it can promote antioxidant formation and degrade thermolabile compounds [11,12]. Among dehydration technologies, spray drying is the most widely used method for industrial production of fruit extract powders because of its continuous operation, high efficiency, and relatively low cost [13,14]. Compared with freeze-drying, which is expensive and time-consuming [15], convective and infrared drying are more suitable for drying intact fruits or solid tissues than for producing powders from liquid extracts. The addition of maltodextrin improves powder recovery, encapsulates bioactive compounds, and enhances storage stability. However, the high inlet temperatures used during spray drying may affect physicochemical properties and antioxidant capacity, highlighting the need to optimize processing conditions.

The interaction between maltodextrin concentration and inlet temperature during spray drying has been evaluated for its effect on the retention of phenolic compounds, flavonoids, and overall antioxidant activity, as well as their stability during extended storage [16,17]. Maltodextrin is an appropriate carrier agent due to its versatility, thermal stability, and effectiveness in preserving bioactive compounds and enhancing bioavailability [18]. Moreover, spray drying offers advantages including scalability for industrial production, cost-effectiveness, and improved stability of heat-sensitive materials [19]. The most common spray-drying conditions for producing fruit and vegetable powders are inlet air temperatures of 120–180 °C and maltodextrin concentrations of 7–20% [20].

Antioxidant assays such as FRAP, ABTS, and DPPH are commonly used to evaluate the antioxidant capacity of fruit-derived products, providing comprehensive insights into their reducing power and free-radical scavenging activity [21]. These antioxidant parameters can also serve as indicators for determining stability during storage, highlighting the need for evaluation of processing conditions to optimize product quality and shelf life [22,23].

Previous studies on spray-dried fruit powders have mainly focused on fresh or minimally processed materials. In contrast, this study investigated a thermally aged fruit matrix that had already undergone non-enzymatic browning before spray drying. To our knowledge, limited information is available on converting this pre-aged material into a stable bioactive food ingredient. We hypothesized that spray drying with appropriate carriers and process conditions could yield a powder with acceptable physicochemical properties and reasonable storage stability. Therefore, this study aimed to (1) optimize the spray-drying conditions for black lychee extracted powder (BLEP) by evaluating the effects of maltodextrin concentration (5–30%) and inlet temperature (160–200 °C) using a 2^2^ factorial design, and (2) assess the physicochemical characteristics and antioxidant stability of the optimized powder during six-month storage at different temperatures. The selected factor ranges were intentionally extended beyond those commonly reported in the literature to define the practical operating limits for this pre-aged material.

## 2. Materials and Methods

### 2.1. Materials and Chemicals

Black lychee (*Litchi chinensis* Sonn. var. Hong Huay) was produced following an optimized condition protocol by a previous study [9]. Fresh lychees from Chiang Mai province, Thailand, during its cultivation period in April–May 2025, were collected and dried (at 60 °C for 48 h through hot-air dryer) to obtain dried lychee pulp (moisture around 15–18%). The dried lychee samples were stored at −18 °C until further analysis.

The chemical substances, reagents, and standards applied in this research included 3,5-Dinitrosalicylic acid (DNS) 98% (Sigma-Aldrich, Bangalore, India), 2,2-Diphenyl-1-picrylhydrazyl (DPPH) (Sigma-Aldrich, India), Trolox (Sigma-Aldrich, Buchs, Switzerland), 2,4,6-Tris(2-pyridyl)-s-triazine (TPTZ) (Sigma-Aldrich, Buchs, Switzerland), Gallic acid 99% (Sigma-Aldrich, St. Louis, MO, USA), O-Phthaldialdehyde (OPA) (Sigma-Aldrich, Shanghai, China), Phenol detached crystals 99.5% (Loba Chemie, Mumbai, India), Ferric chloride hexahydrate (FeCl_3_ · 6H_2_O) (Loba Chemie, India), Potassium persulfate (Loba Chemie, India), di-Sodium tetraborate decahydrate (Borax) AR/ACS (Loba Chemie, India), Sodium dodecyl sulfate (SDS) (Loba Chemie, India), Sodium hypochlorite (Loba Chemie, India), Boric acid (Loba Chemie, India), Sodium acetate trihydrate (KemAus, Sydney, Australia), Ferrous Sulfate Heptahydrate (FeSO_4_ · 7H_2_O) (QReC, Auckland, New Zealand), 2,2′-Azino-bis(3-ethylbenzothiazoline-6-sulfonic acid) (ABTS) (Sigma-Aldrich, China), Sodium carbonate (Na_2_CO_3_) (KemAus, Sydney, Australia), D-glucose (Unilab, Springvale, Australia), Folin–Ciocalteu’s phenol (Merck, Darmstadt, Germany), 2-Mercaptoethanol (Merck, Germany), N-acetyl-L-cysteine (Merck, Germany), and L-lysine monohydrochloride (HiMedia, Mumbai, India); Sodium hydroxide, Acetic acid glacial, HPLC Acetonitrile, HPLC Methanol, Ethanol AR, Hexane AR, Methanol AR, Hydrochloric acid 37% AR, Sulfuric acid 98%, Potassium sulfate and Sodium citrate tribasic dihydrate were purchased from RCI Labscan, Thailand. A set of 17 analytical amino acid standards were purchased (Wako Pure Chem. Co., Osaka, Japan). Food-grade maltodextrin was supplied by Banpong Novitat (Ratchaburi, Thailand). All solutions were prepared using ultrapure demineralized water from a Zeneer UP 900 water purification system (Seoul, Republic of Korea).

### 2.2. Black Lychee Production and Extract Preparation

Black lychee was prepared according to the moist-dry heating method described by Somjai et al. [24] with some modifications. Whole dried lychee fruits were thermally aged at 70 °C for 20 days in a sealed desiccator containing a saturated NaCl solution to maintain a relative humidity of 75%. The desiccator was placed in an incubator (Daihan Scientific, Wonju, Republic of Korea) throughout the aging period. After aging, the peel and seed were removed, and 50 g of black lychee pulp was homogenized with distilled water at a ratio of 1:10 (*w*/*v*) using a Sonics Vibra-Cell ultrasonic processor (Model VCX-750, Newtown, CT, USA) operated at 28% amplitude for 15 min. The homogenate was filtered through a 100 μm mesh to remove insoluble materials. The extracted solution was transferred into amber bottles and stored at −18 °C until further analysis.

### 2.3. BLEP Preparation

The production of BLEP was optimized by evaluating the effects of maltodextrin concentration (5–30%) and inlet temperature (160–200 °C) using a 2^2^ factorial design with two center points as shown in Table 1. The six experiments constitute a 2^2^ full factorial design with two replicated center points. The four factorial experiments are the corner combinations of maltodextrin (5 and 30%) and inlet temperature (160 and 200 °C) and experiments 5–6 were replicated center points (17.5%, 180 °C) used to estimate pure error and to test for curvature. The factor ranges were chosen to bracket the levels commonly reported for fruit and vegetable powders (7–20% maltodextrin; 120–180 °C), then extended at both ends to capture the boundaries of feasible operation for this design. The black lychee extracted samples were mixed with maltodextrin and heated to 60 °C with continuous stirring until completely dissolved. Spray drying was performed using a BUCHI Mini Spray Dryer B-290 (BUCHI, Bangkok, Thailand). The aspirator, pump, and nozzle were set at 85%, 20%, and 4, respectively. The resulting BLEP samples were collected and transferred to polyethylene bags and stored at −18 °C until further analysis.

### 2.4. Assessment of Color Parameters, Moisture Content, and Powder Yield of BLEP

The physical properties of BLEP, including color value, moisture content, and powder yield, were evaluated. Color measurements were performed using a colorimeter (CR-400, Konica Minolta, Japan). The results were expressed in the CIE L*a*b* color space, where L* represents lightness, a* indicates the red–green axis, and b* denotes the yellow–blue axis [25]. Moisture content was determined according to the AOAC standard method [26]. Powder yield (%) was calculated as the ratio of the weight of the powder recovered after spray drying to the initial weight of the feed solution, according to Equation (1):

(1) $$\mathrm{Powder}\;\mathrm{yield}\;(\%)=\frac{W_{sample}}{W_{feed}}\times 100$$

where $W_{sample}$ is the weight of the collected powder (g) and $W_{feed}$ is the initial weight of the feed solution (g) [27].

### 2.5. Scanning Electron Microscopy (SEM) Analysis of BLEP

The surface morphology of the optimaized BLEP sample was examined using SEM (JSM-IT200, JEOL Ltd., Tokyo, Japan). The samples were freeze-dried and mounted on aluminum stubs using double-sided conductive carbon tape and sputter-coated with a thin layer of gold. SEM observations were performed at a voltage of 5–10 kV. Micrographs were acquired at a magnification of 1000× to evaluate surface structure, particle morphology, and porosity.

### 2.6. Chemical Composition Analysis of BLEP

#### 2.6.1. HPLC-Based Profiling of Individual Phenolic Compounds and Spectrophotometric Determination of Total Phenolic and Flavonoid Contents

All BLEP were analyzed using an HPLC system (Shimadzu, Kyoto, Japan) equipped with a Prominence diode array detector (DAD) and a C18 column (250 × 4.6 mm, GL Sciences, Torrance, CA, USA). Separation was carried out using a binary gradient system consisting of mobile phase A (2% acetic acid in water) and mobile phase B (100% acetonitrile) at a flow rate of 1 mL min^−1^, with a total run time of 85 min. The column temperature was maintained at 30 °C, and the gradient program followed the method of Chaipoot et al. [28]. For sample preparation, BLEP the sample was diluted with distilled water. A total of 500 µL of sample was mixed with 500 µL of absolute acetonitrile, vortexed, and filtered through a 0.45 µm membrane filter prior to analysis. An injection volume of 10 µL was used and detection was performed at 280 nm. Identification and quantification were conducted using a mixed standard solution containing 19 phenolic compounds, comprising gallic acid, theobromine, protocatechuic acid, *p*-hydroxybenzoic acid, catechin, chlorogenic acid, caffeine, vanillic acid, caffeic acid, syringic acid, epicatechin, vanillin, *p*-coumaric acid, ferulic acid, sinapic acid, rutin, myricetin, quercetin, and *trans*-cinnamic acid.

Hydroxymethylfurfural (HMF) was determined using the same HPLC-DAD system according to the OIV official HPLC method with slight modifications [29]. Chromatographic separation was carried out at 40 °C using gradient elution with mobile phase A (10% methanol), mobile phase B (deionized water), and mobile phase C (80% methanol) at a flow rate of 1.0 mL min^−1^. Samples were filtered through a 0.45 µm membrane filter before analysis. A 10 µL injection volume was used, HMF was detected at 280 nm, and quantification was performed by standard calibration. The results were expressed as g of HMF per kilogram of sample.

Total phenolic content (TPC) and total flavonoid content (TFC) were determined following the method described by Zhang et al. [30], with minor modifications. BLEP was diluted with distilled water prior to analysis. TPC was measured using the Folin-Ciocalteu colorimetric method and expressed as milligrams of gallic acid equivalents (GAE) per 100 g of sample on a dry weight basis, while TFC was determined using the aluminum chloride colorimetric method and expressed as milligrams of quercetin equivalents (QE) per 100 g of sample on a dry weight basis.

#### 2.6.2. Quantification of Total Sugars, Reducing Sugars, and Individual Monosaccharides

The total sugar content was determined using the colorimetric phenol–sulfuric acid method described by DuBois et al. [31] with some modifications. The sample was diluted with distilled water and mixed with 250 µL of 5% (*w*/*v*) phenol solution, followed by the addition of 1.25 mL of 96% sulfuric acid, and placed in the dark for 30 min before the absorbance was measured using UV-vis spectroscopy (UV1800; Shimadzu, Kyoto, Japan) at 490 nm. The blank was prepared using distilled water and subjected to the same procedure as the samples. A standard curve using D-glucose (concentration range: 0.025–0.200 mg/mL) was used.

Reducing sugar content was determined using a modified 3,5-dinitrosalicylic acid (DNS) method based on Gandhi et al. [32]. Glucose solutions (0.1–5 mg/mL) were used to construct a standard calibration curve, and crude extracts were appropriately diluted prior to analysis. A total of 1 mL of sample was mixed with 4 mL of DNS reagent, covered to minimize evaporation, and heated at 90 °C for 5 min. After rapid cooling in an ice bath, 10 mL of distilled water was added to stabilize color development. A blank was prepared using distilled water and treated under identical conditions. Absorbance was measured at 550 nm using a UV-Vis spectrophotometer (UV-1800; Shimadzu, Japan).

Monosaccharide composition, including allulose, fructose, mannose, and glucose, was determined by high-performance liquid chromatography (HPLC) following the method of Somjai et al. [24]. All analyses were performed on BLEP samples, and results are expressed on a dry-weight basis (db).

#### 2.6.3. Post-Column Amino Acid Analysis

The analysis of 17 amino acids was performed using high-performance liquid chromatography (HPLC) with post-column derivatization. Separation was achieved using a Shim-pack Amino-Na column (100 mm × 6.0 mm i.d., 5 μm; P/N: 228-18837-91, Shimadzu, Japan) coupled with a Prominence RF-20A fluorescence detector (Shimadzu, Japan). The mobile phase system consisted of three Na-type eluents: mobile phase A (sodium citrate buffer, pH 3.23), mobile phase B (sodium citrate buffer, pH 10.0), and mobile phase C (0.2 M sodium hydroxide). The chromatographic conditions were set at a column temperature of 60 °C, a flow rate of 0.4 mL min^−1^, and an injection volume of 10 μL. A mixed standard solution containing 17 amino acids, including alanine (Ala), arginine (Arg), aspartic acid (Asp), cysteine (Cys), glutamic acid (Glu), glycine (Gly), histidine (His), isoleucine (Ile), leucine (Leu), lysine (Lys), methionine (Met), phenylalanine (Phe), proline (Pro), serine (Ser), threonine (Thr), tyrosine (Tyr), and valine (Val), was used for identification and quantification. Amino acids were derivatized using O-phthaldialdehyde in the presence of N-acetyl-L-cysteine to enable fluorescence detection under Na-type hydrolysis conditions.

#### 2.6.4. Evaluation of Antioxidant Activity Using FRAP, ABTS, and DPPH Assays

Different antioxidant assays were used to determine the in vitro chemical antioxidant capacity of the samples, following our previous study. The ferric reducing antioxidant power (FRAP) assay was used to assess the reducing ability of antioxidants by measuring their capacity to convert Fe^3+^ to Fe^2+^, reflecting electron-donating potential. The ABTS radical cation decolorization assay was applied to evaluate the ability of antioxidants to scavenge the ABTS radical, which is applicable to both hydrophilic and lipophilic compounds. The DPPH assay was used to determine free radical scavenging activity through a mixed mechanism involving both electron transfer and hydrogen atom transfer. The results of antioxidant activity were expressed in different equivalent forms depending on the assay principle: FRAP values were reported as milligrams of FeSO_4_ equivalents, and ABTS and DPPH values were reported as milligrams of Trolox equivalents (TE), all standardized per 100 g dry weight of the extract.

### 2.7. Stability of Physical and Chemical Properties of BLEP

#### 2.7.1. Storage Stability Study of Optimized BLEP

The storage stability of the optimized BLEP was evaluated over six months at four storage temperatures (4, 25, 35, and 45 °C). Refrigerated storage (4 °C) was included as a low-temperature reference to minimize temperature-induced degradation and to provide a baseline for comparison with ambient (25 °C) and accelerated storage conditions (35 and 45 °C). Samples were sealed in aluminum foil bags and stored under each temperature condition in the dark to minimize light-induced degradation. Physical properties, including color parameters (L*, a*, b*, C*, and H°) and moisture content, as well as chemical properties, comprising TPC, total sugar content, reducing sugar content, the degree of glycation (DG), and antioxidant activity assessed by FRAP, DPPH, and ABTS assays, were measured at monthly intervals over the six-month storage period. Statistical differences among storage temperatures and time points were evaluated by one-way analysis of variance (ANOVA) followed by Tukey’s HSD post hoc test, with significance set at *p* ≤ 0.05.

The relationships among TPC, antioxidant activity (FRAP, DPPH, and ABTS), and storage duration were examined using Pearson correlation analysis (*p* < 0.01). TPC degradation was described by zero-order and first-order kinetic models, with model selection based on adjusted R^2^, and the L_50_ value calculated from the best-fit model.

#### 2.7.2. Determination of the Degree of Glycation (DG)

DG was assessed from the availability of free amino groups using the O-phthaldialdehyde (OPA) assay [24]. Absorbance was measured at 340 nm with a UV−Vis spectrophotometer, and the free amino group content was expressed as milligrams of lysine equivalents per 100 g dry basis. DG was then calculated as the percentage reduction in free amino groups relative to control (Month 0).

### 2.8. Statistical Analysis

All experiments are performed in triplicate, and results are expressed as mean ± standard deviation (SD). Differences among experimental treatments were evaluated by one-way analysis of variance (ANOVA). When a significant effect was detected, mean comparisons were performed using Tukey’s Honest Significant Difference (HSD) post hoc test at a significance level of *p* ≤ 0.05. Response surface analysis and process optimization were performed only for statistically significant responses using Design-Expert software (version 12; Stat-Ease Inc., Minneapolis, MN, USA). The optimum spray-drying condition was identified graphically using an overlay plot of the significant response variables.

To evaluate the degradation kinetics of the quality attributes during storage, zero- and first-order kinetic models were fitted by linear regression using SPSS software (version 17.0; SPSS Inc., Chicago, IL, USA). For the zero-order model, the measured quality parameter ($C_{t}$) was directly regressed against storage time (t) according to Equation (2):

(2) $$C_{t}=C_{0}-kt$$

where $C_{t}$ is the value of the quality parameter at storage time t, $C_{0}$ is the initial value, and k is the zero-order degradation rate constant.

For the first-order model, the natural logarithm of the quality parameter was regressed against storage time according to Equation (3):

(3) $$ln\left(C_{t}\right)=ln\left(C_{0}\right)-kt$$

where k is the first-order degradation rate constant.

The kinetic model providing the higher Adjusted $R^{2}$ was selected as the most appropriate model for describing the degradation behavior.

## 3. Results and Discussion

### 3.1. Effects of Maltodextrin Content and Inlet Temperature on Physicochemical and Antioxidant Qualities of BLEP

#### 3.1.1. Color Characteristics, Moisture Content, and Powder Yield of BLEP

The physical properties of BLEP produced under varying spray-drying conditions are represented in Table 2. Lightness (L*) varied from 59.26 to 75.61, with the maximum value attained at 30% maltodextrin and 200 °C inlet temperature. The minimum L* value was recorded at 5% maltodextrin and 200 °C inlet temperature, corresponding to a darker powder appearance. The highest redness (a*) values were observed at 5% maltodextrin with inlet temperatures of 200 °C and 160 °C (a* = 12.30 and 12.26, respectively), while the lowest was recorded at 30% maltodextrin and 200 °C (a* = 8.87). Yellowness (b*) and chroma (C*) were greatest at 5% maltodextrin and 160 °C inlet temperature, reaching 22.02 and 26.46, respectively, indicating higher color saturation. The hue angle (H°) ranged from 59.71 to 64.76, with the maximum observed at 30% maltodextrin and 200 °C inlet temperature.

Moisture content ranged from 0.08% to 5.08%. The highest moisture contents were observed at 5% maltodextrin (5.08% at 160 °C and 4.48% at 200 °C), while the lowest values were found at 17.5% maltodextrin (0.09% and 0.08%). Powder yield ranged from 41.33% to 64.73%, with the lowest yield at 5% maltodextrin and 200 °C and the highest at the center-point condition (17.5% maltodextrin and 180 °C). Overall, maltodextrin concentration was the main factor affecting both responses. Increasing the maltodextrin level reduced powder stickiness and moisture content by increasing the feed solids content and glass transition temperature. However, powder yield did not continue to increase at the highest maltodextrin level, suggesting that an intermediate concentration (17.5%) provided the best powder recovery under the conditions tested. The residual moisture content of BLEP was inversely related to powder yield. Powders produced with 5% maltodextrin had higher moisture contents (4.48–5.08%) and lower yields (41.33–52.00%), whereas those containing 17.5–30% maltodextrin showed lower moisture contents (0.08–0.83%) and higher yields (57.00–64.73%). This trend can be explained by the plasticizing effect of residual water, which depresses the glass transition temperature (Tg) of the particle surface, increasing stickiness and wall deposition during spray drying, which reduces powder recovery [33]. Increasing the maltodextrin concentration increases the feed solid content and Tg, promoting moisture removal and reducing stickiness, thereby minimizing wall deposition and improving powder yield [34,35].

#### 3.1.2. Chemical Properties and Antioxidant Activities of BLEP

The chemical properties of BLEP produced under varying spray-drying conditions are presented in Table 3. Total sugar content ranged from 46.82 to 59.18 g glucose/100 g db. The highest values were obtained at 5% maltodextrin with inlet temperatures of 200 °C and 160 °C (59.18 and 59.13 g glucose/100 g db, respectively), whereas the lowest value was observed at the center-point condition. Reducing sugar content ranged from 29.03 to 51.89 g glucose/100 g db, with the highest value at 5% maltodextrin and 160 °C, followed by 5% maltodextrin and 200 °C (47.96 g glucose/100 g db), while the lowest value occurred at 30% maltodextrin and 200 °C.

Total phenolic content (TPC) ranged from 396.02 to 1203.52 mg GAE/100 g db. The highest TPC values were observed at 5% maltodextrin with inlet temperatures of 160 °C and 200 °C (1203.52 and 1124.86 mg GAE/100 g db, respectively), whereas the lowest value was found at 30% maltodextrin and 200 °C. Similarly, total flavonoid content (TFC) ranged from 344.12 to 1672.51 mg QE/100 g db, with the highest value at 5% maltodextrin and 200 °C (1672.51 mg QE/100 g db), followed by 5% maltodextrin and 160 °C (1544.14 mg QE/100 g db). TPC and TFC are expressed using different calibration standards and therefore are not directly comparable quantitatively.

Antioxidant capacity varied in the same direction across the design. DPPH radical scavenging activity ranged from 230.40 to 691.67 mg TE/100 g db, ABTS radical scavenging activity from 671.83 to 1560.52 mg TE/100 g db, and FRAP from 707.78 to 2215.19 mg FeSO_4_/100 g db; in each assay the maximum occurred at 5% maltodextrin and 160 °C. The minimum was recorded at 30% maltodextrin and 200 °C for DPPH and FRAP, and at 30% maltodextrin and 160 °C for ABTS. Overall, lower maltodextrin concentrations resulted in higher sugar, phenolic, flavonoid, and antioxidant contents. Since maltodextrin does not contribute to measurable phenolic compounds or antioxidant activity, increasing its concentration dilutes the extract-derived bioactive compounds on a dry-weight basis.

#### 3.1.3. Sugar and Phenolic Profiles of BLEP by HPLC

The monosaccharide composition, including rare sugars (allulose) and major sugar components (fructose, mannose, and glucose), is presented in Table 4. Consistent with the known sugar profile of lychee-derived ingredients, fructose and glucose were identified as the dominant monosaccharides across all spray-drying conditions from previous research. The highest concentrations of sugar were recorded at 5% maltodextrin under inlet temperatures of 160 °C and 200 °C, with fructose reaching 6.36 and 5.33 g/100 g db and glucose reaching 12.62 and 10.21 g/100 g db, respectively. Mannose followed the same trend, with the highest levels at 5% maltodextrin under 160 °C and 200 °C (0.87 and 0.79 g/100 g db, respectively). In contrast, the lowest concentrations of fructose and glucose were observed at 30% maltodextrin under both inlet temperatures. Notably, allulose was detected in the BLEP samples. Allulose, a rare C-3 epimer of fructose known for its low caloric value and potential functional properties, was most abundant at 5% maltodextrin and 200 °C inlet temperature (0.50 g/100 g db), followed by 5% maltodextrin and 160 °C inlet temperature (0.39 g/100 g db). The appearance of allulose at lower maltodextrin concentrations and higher inlet temperatures suggests that thermal processing conditions may promote the epimerization of fructose to allulose, consistent with the temperature-dependent sugar isomerization observed in dried lychee pulp in previous research. The detection of these rare sugars in spray-dried BLEP highlights its potential as a functional food ingredient.

HPLC analysis identified seven phenolic compounds in BLEP samples, comprising protocatechuic acid, catechin, caffeine, vanillic acid, epicatechin, rutin, and *trans*-cinnamic acid. Among these, vanillic acid was the most abundant, peaking at 5% maltodextrin and 200 °C (362.99 mg/100 g db). Caffeine, epicatechin, catechin, and rutin also reached their highest levels at 5% maltodextrin and 160 °C inlet temperature, while protocatechuic acid reached its highest concentration at 5% maltodextrin and 200 °C (8.17 mg/100 g db). As a result, spray-drying at 5% maltodextrin under both 160 °C and 200 °C inlet temperatures consistently yielded the highest concentrations of both monosaccharides and individual phenolic compounds.

#### 3.1.4. Amino Acid Profile of BLEP

The amino acid composition of BLEP produced under varying maltodextrin concentrations and inlet temperatures is presented in Table 5. Total amino acid content was highest at 5% maltodextrin under 160 °C and 200 °C (127.89 and 122.72 mg/100 g db, respectively), with leucine as the predominant amino acid (83.35 and 85.42 mg/100 g db), followed by alanine, valine, and methionine. Threonine, cysteine, isoleucine, tyrosine and histidine were not detected in any sample, and phenylalanine was detected only at 5% maltodextrin. Glutamic acid and aspartic acid were most concentrated under 5% maltodextrin conditions, while arginine showed an opposing trend, peaking at 30% maltodextrin and 200 °C (8.39 mg/100 g db). The predominance of leucine and the presence of glutamic and aspartic acid describe the compositional profile of the powder. The relatively low total free amino acid content is consistent with partial consumption of free amino groups through Maillard conjugation during thermal ageing.

#### 3.1.5. Response Surface Methodology (RSM) and Optimization Conditions

**Figure 1:**
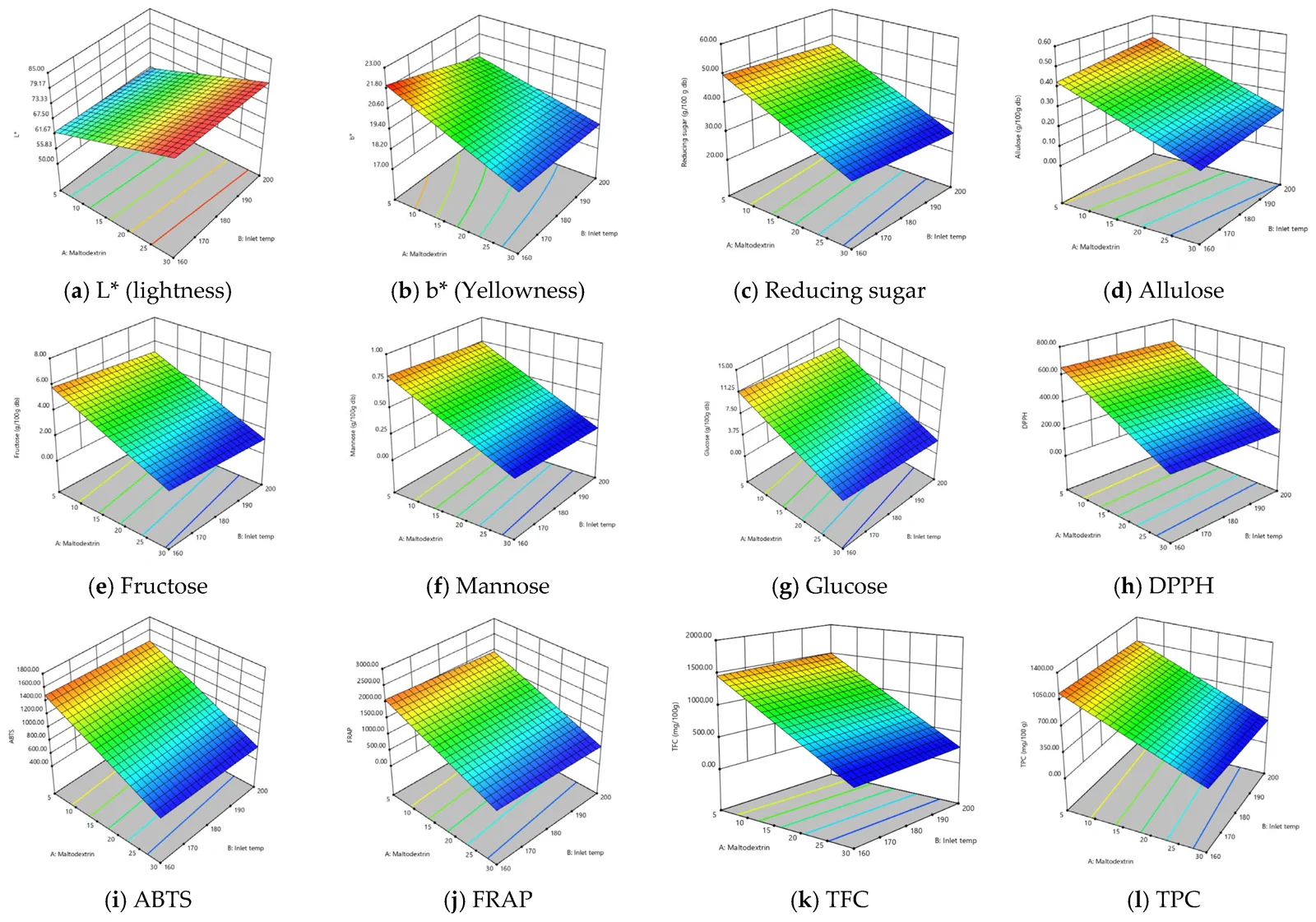

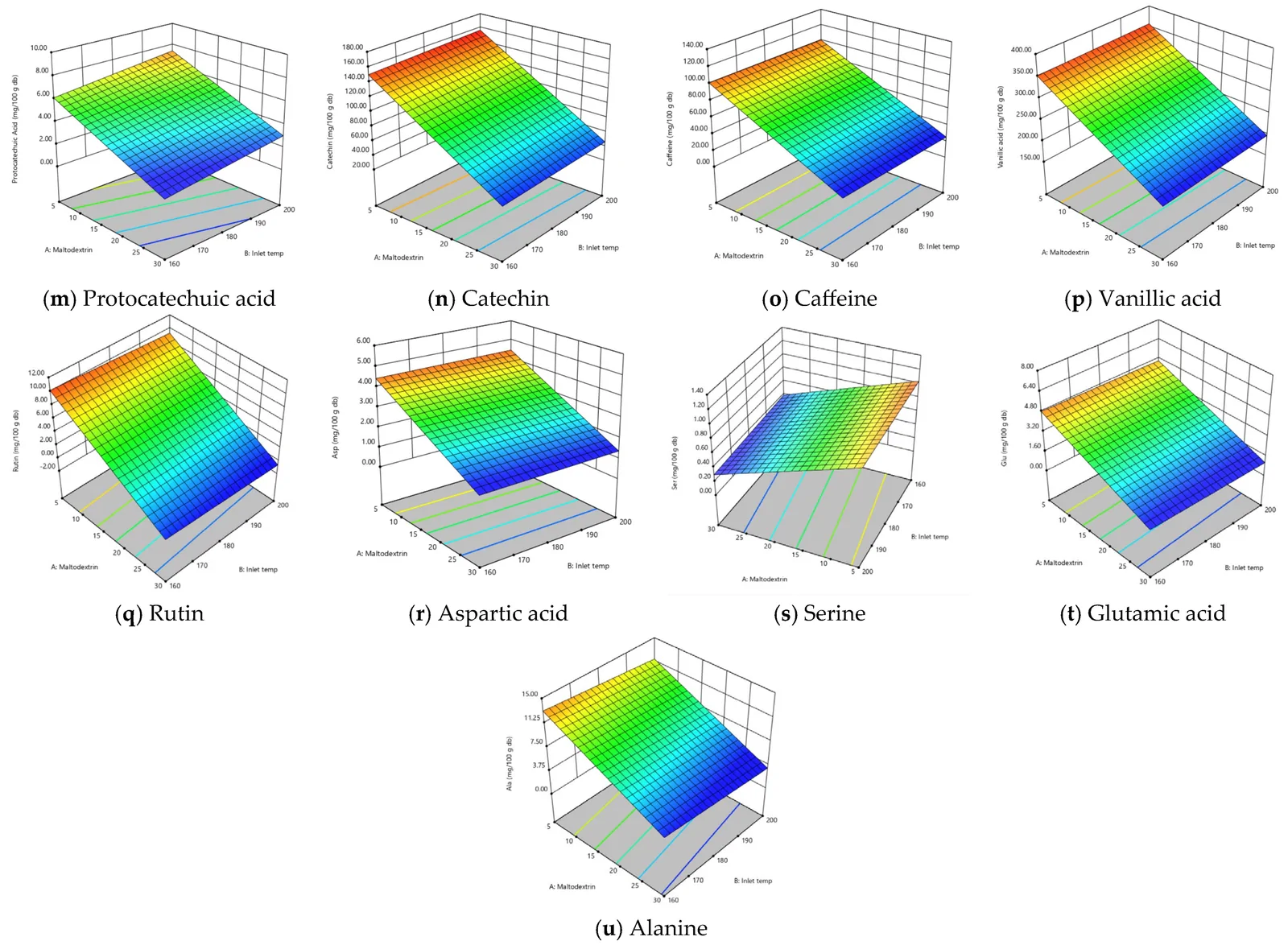
Three-dimensional response surface plots illustrating the effects of maltodextrin concentration (A, %) and inlet temperature (B, °C) on the physicochemical and bioactive properties of BLEP: (**a**) lightness (L*); (**b**) b* (Yellowness); (**c**) reducing sugar; (**d**) allulose; (**e**) fructose; (**f**) mannose; (**g**) glucose; (**h**) DPPH; (**i**) ABTS; (**j**) FRAP; (**k**) TFC; (**l**) TPC; (**m**) protocatechuic acid; (**n**) catechin; (**o**) caffeine; (**p**) vanillic acid; (**q**) rutin; (**r**) aspartic acid; (**s**) serine; (**t**) glutamic acid; (**u**) alanine.

Response surface methodology (RSM) was applied to evaluate the combined effects of maltodextrin concentration (A) and inlet temperature (B) on multiple quality attributes of BLEP. Regression equations and model fit statistics are presented in Table 6. The developed regression models showed good fit, with significant (*p* ≤ 0.05) effects and satisfactory adjusted R^2^ values (0.77–0.98). The lack-of-fit was not significant, indicating that the models adequately described the experimental responses. The three-dimensional response surface plots (Figure 1) illustrate the effects of maltodextrin concentration and inlet temperature on the measured responses. In most models, maltodextrin concentration was the dominant factor, whereas inlet temperature had a comparatively smaller effect, except for L* and serine. Regression analysis (Table 6) also confirmed that maltodextrin concentration was the primary significant factor affecting most responses.

The optimum spray-drying condition was identified graphically using the overlay plot function in Design-Expert v12. No numerical desirability function or response weighting was applied. Significant responses, including sugar components (allulose, fructose, mannose, glucose, and reducing sugar), antioxidant capacity (DPPH, ABTS, and FRAP), TPC, TFC, and amino acids (aspartic acid, serine, glutamic acid, and alanine), were set to be maximized, while the color parameters (L* and b*) were constrained within the experimental range. The overlap of these criteria defined the feasible operating region, from which 17.80% maltodextrin and an inlet temperature of 200 °C were selected as the optimum condition.

The optimum was chosen by balancing all responses rather than maximizing bioactive compounds alone. Although 200 °C was the highest inlet temperature tested, the regression models showed that inlet temperature had a smaller influence on most bioactive compounds than maltodextrin concentration. Moreover, several responses, including allulose, TFC, protocatechuic acid, catechin, vanillic acid, and aspartic acid, increased with higher inlet temperature. Thus, operating at 200 °C maintained bioactive compound levels while improving drying efficiency, making it a practical condition for scale-up.

#### 3.1.6. Morphological Characterization of BLEP Using Scanning Electron Microscopy (SEM)

**Figure 2:**
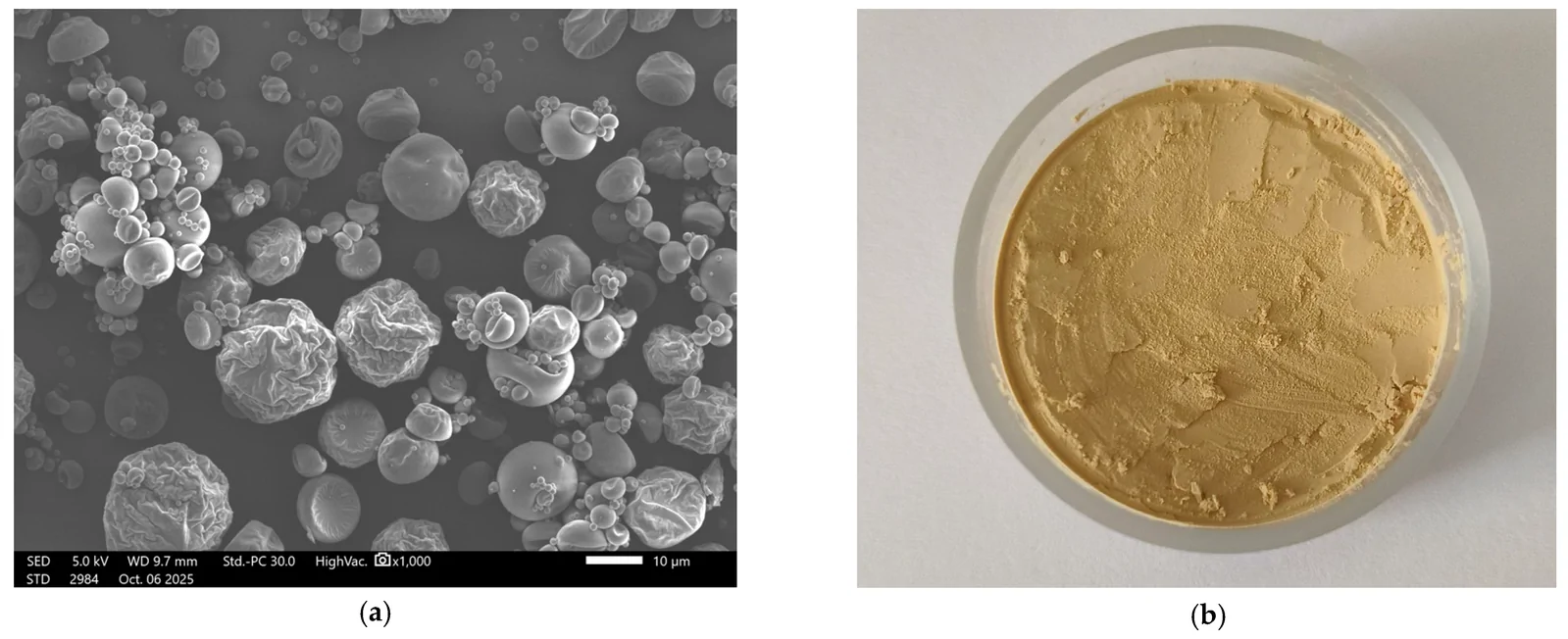
SEM micrographs (1000×) of (**a**) lychee incubated at 70 °C for 20 days and (**b**) spray-dried BLEP.

The surface morphology was investigated using SEM as shown in Figure 2. The incubated lychee exhibited a smooth, continuous sheet-like structure with evident shrinkage and folding of the matrix (Figure 2a), indicating structural contraction caused by moisture loss during the incubation process. This dehydration-induced collapse resulted in a dense structure with low porosity. In contrast, Figure 2b illustrates the morphology of the BLEP, which consisted of discrete, spherical particles with good dispersion. The particle surfaces were rough, with some showing partial collapse or wrinkling, characteristics associated with spray-dried materials. The presence of maltodextrin as a carrier contributed to the formation and stabilization of these particles.

### 3.2. Storage Stability of Physicochemical Properties in the Optimized Conditions of BLEP

#### 3.2.1. Physical Stability Study of the Optimized-Condition BLEP

The changes in color parameters (L*, a*, b*, C*, and H°) and moisture content of optimized BLEP during six months of storage at 4, 25, 35, and 45 °C are presented in Table S1. All color parameters remained highly stable throughout the storage period across all temperature conditions. Lightness (L*) was maintained within a narrow range of 75.07–76.65 throughout six months, indicating negligible browning development during storage. Similarly, redness (a*), yellowness (b*), chroma (C*), and hue angle (H°) showed only minor fluctuations across all time points and temperatures, with b* ranging from 20.48 to 22.13 and H° remaining consistently within 66.37–67.27°. This color stability is consistent with findings reported for other spray-dried tropical fruit powders, where maltodextrin encapsulation has been shown to protect chromatic parameters over extended storage periods [7,36]. The minimal variation in L* throughout the study period suggests that Maillard browning reactions had reached completion prior to powder production, rendering the residual color resistant to further thermal degradation within the storage temperature range investigated.

Moisture content decreased progressively during storage at all temperatures, declining from 1.13% at month 0 to below 0.1% by month 5 and remaining at this level through month 6. This reduction is likely due to the amorphous maltodextrin matrix, which immobilizes free water and limits moisture migration over time [7]. The similar moisture loss across all storage temperatures suggests that this change was mainly governed by the physicochemical properties of the maltodextrin matrix rather than storage temperature. Water activity was not measured in this study because the moisture content fell below 0.1%, making reliable determination impractical.

#### 3.2.2. Chemical Stability of BLEP During Storage

**Figure 3:**
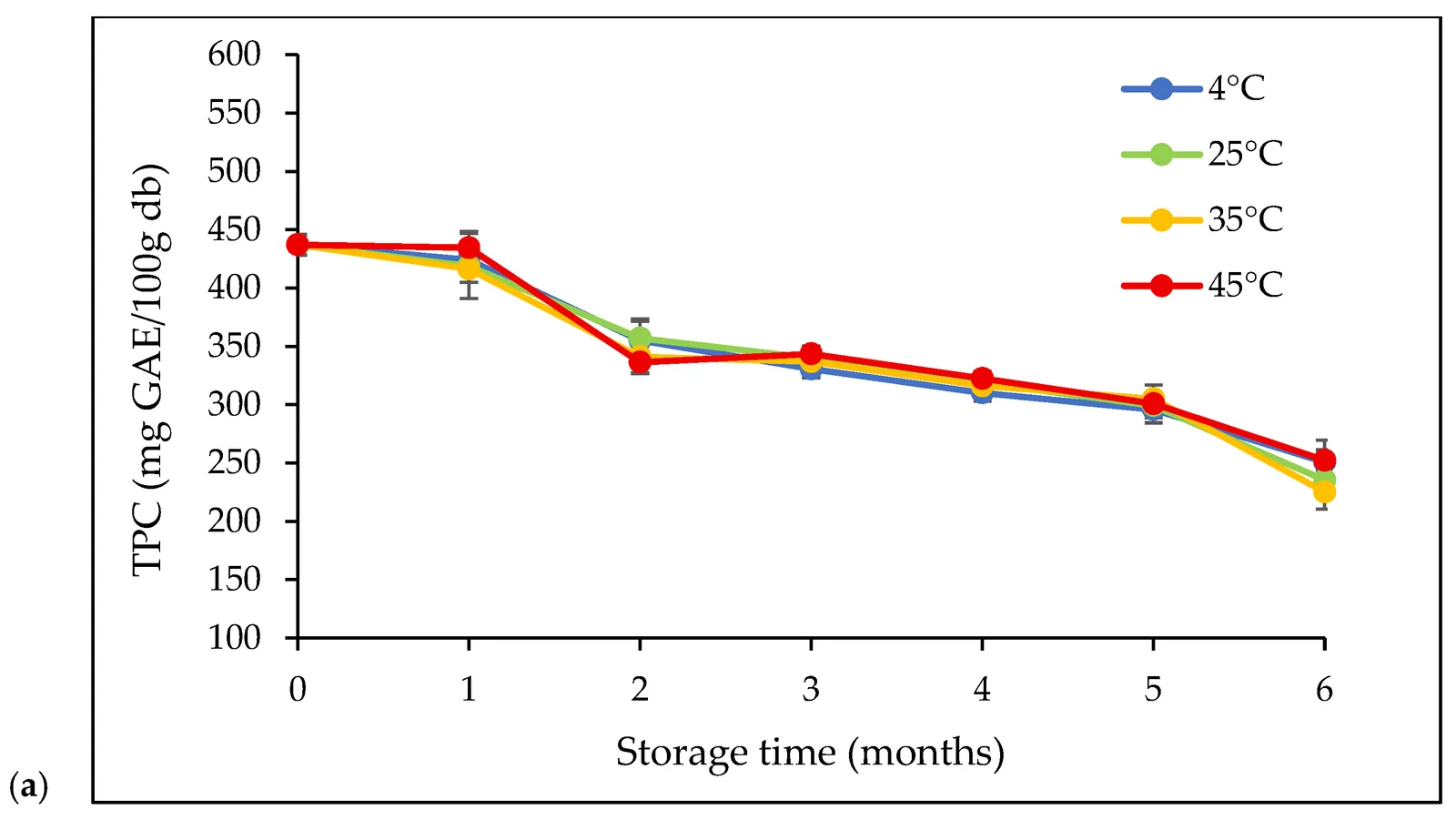

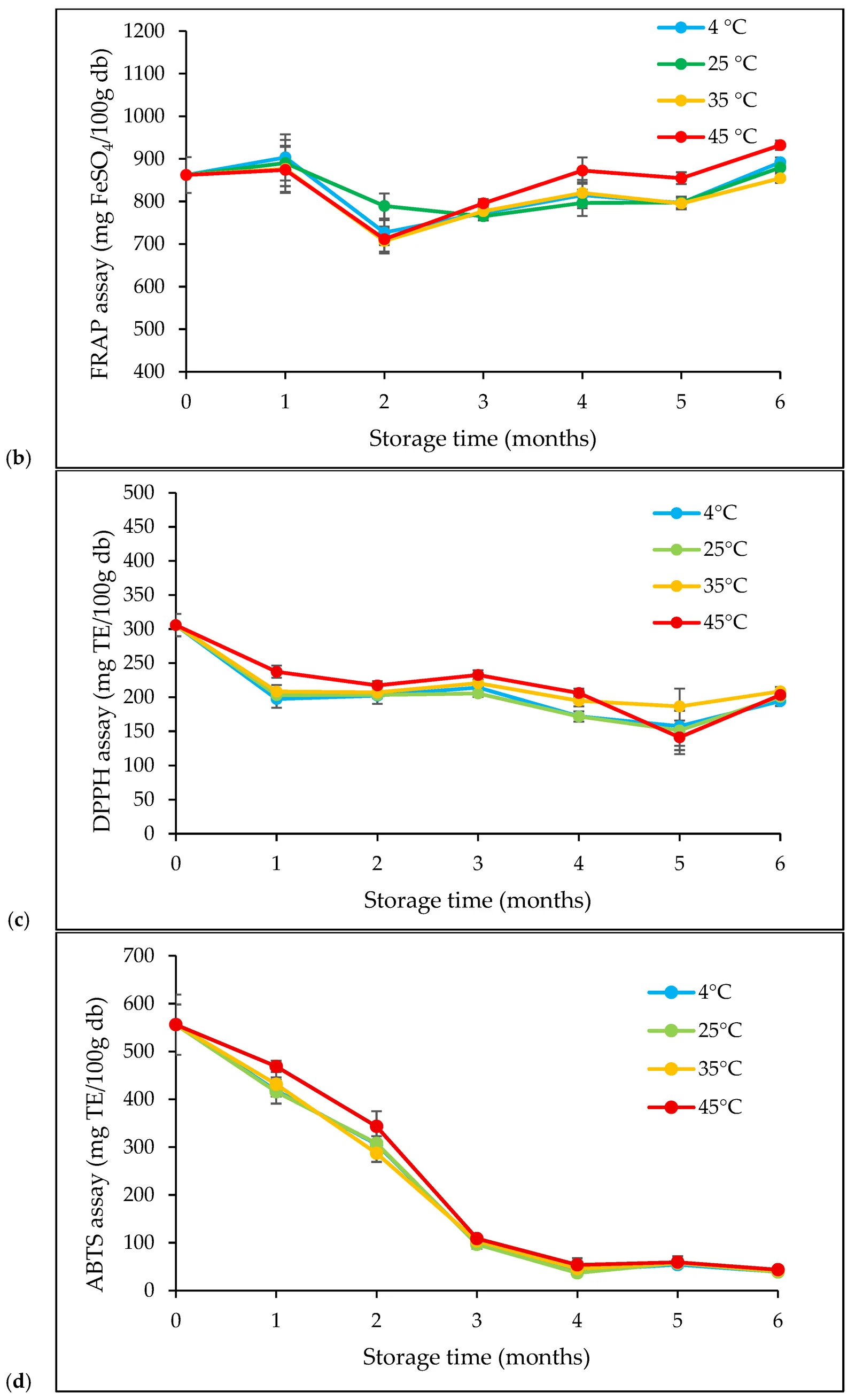
Changes in chemical stability parameters of black lychee extracted powder (BLEP) during six months of storage at 4, 25, 35, and 45 °C: (**a**) TPC, (**b**) FRAP, (**c**) DPPH, and (**d**) ABTS. All values are expressed on a dry-weight basis (db). Data are presented as mean ± SD (*n* = 3).

The chemical stability of optimized BLEP was evaluated by monitoring TPC, antioxidant capacity (DPPH, ABTS, and FRAP), total sugar content, reducing sugar content, and degree of glycation (DG) at monthly intervals over six months at 4, 25, 35, and 45 °C, as presented in Tables S2 and S3, and Figure 3.

The results show that TPC declined progressively under all storage conditions, from 437.11 mg GAE/100 g db at month 0 to 416–434 mg GAE/100 g db at month 1, continuing to 225–252 mg GAE/100 g db by month 6.

The three antioxidant assays exhibited divergent degradation patterns. DPPH and ABTS respond principally to hydrogen-donating and electron-transferring species and therefore track the loss of accessible phenolic hydroxyl groups, whereas FRAP measures single-electron reduction of the Fe^3+^-TPTZ complex and responds to a broader pool of reducing species, including reducing sugars and intermediate reaction products, which were retained or generated during storage. As the results, DPPH activity declined rapidly from 305.71 mg TE/100 g db at month 0 to 141.04–232.57 mg TE/100 g db within the first month, then stabilized through months 2–6 (141–237 mg TE/100 g db), reflecting early consumption of accessible hydrogen-donating scavengers via hydrogen atom transfer and single electron transfer mechanisms [37]. ABTS activity showed a more continuous decline, from 555.98 mg TE/100 g db at month 0 to 415.64–468.73 mg TE/100 g db at month 1, falling sharply to 96.45–108.77 mg TE/100 g db by month 3 and reaching 38.58–43.51 mg TE/100 g db at month 6, reflecting progressive degradation of Maillard conjugate compounds via the sequential proton loss electron transfer mechanism. FRAP values were comparatively stable, declining from 862.06 mg FeSO_4_/100 g db at month 0 to a minimum of 707–789 mg FeSO_4_/100 g db at month 2, before recovering and stabilizing at 765–932 mg FeSO_4_/100 g db through months 3–6, suggesting sustained retention of reducing power compounds within the maltodextrin matrix.

Total sugar content showed initial variability at month 1 (83.69–93.53 g glucose/100 g db) before stabilizing through months 3–6 (93.71–99.07 g glucose/100 g db), indicating total sugar equilibrium during storage. Reducing sugars increased from 35.36 g glucose/100 g db at month 0 to 40.75–46.19 g glucose/100 g db by month 1, likely due to hydrolysis of non-reducing sugars promoted by residual moisture, and remained relatively stable through month 6 (41.03–46.19 g glucose/100 g db). This early increase in reactive carbonyl groups coincided with a rise in degree of glycation (DG), peaking at approximately 22% during months 1–3, before stabilizing or slightly declining through months 4–6 (13.56–22.54%), suggesting that the glycation system approached equilibrium between sugar formation and consumption. Differences in DG among storage temperatures were not consistent across the study period, indicating that Maillard reaction progression in the low-moisture BLEP matrix was not strongly temperature-dependent within the range tested.

HMF is an intermediate marker of the early-to-intermediate stages of the Maillard reaction. The results showed that HMF contents varied within a relatively narrow range (3.35–4.03 g/kg) across all storage temperatures and time points. This limited variation suggests that further progression of the Maillard reaction was constrained under the low-moisture conditions achieved after spray drying. Such suppression of the Maillard reaction beyond the early glycation stages under low moisture and mild temperatures is consistent with previous reports [38]. Overall, these findings indicate that the Maillard reaction system in the black lychee powder remained relatively stable during the six-month storage period under the conditions studied.

#### 3.2.3. Kinetic Modeling and Interrelationship of Antioxidant Capacity and Total Phenolics in BLEP During Storage

Pearson correlation analysis was performed to evaluate the relationships among antioxidant capacity, phenolic content, and storage time. The Pearson correlation analysis revealed strong interdependencies between antioxidant capacity (DPPH, ABTS), TPC and storage time (*p* < 0.01). A strong positive correlation between TPC and ABTS was observed (r = 0.914, *p* < 0.01). Although DPPH also showed a significant correlation with TPC (r = 0.450, *p* < 0.01), the weaker association compared to ABTS may reflect differences in radical accessibility and assay sensitivity. A key observation is the strong negative correlation between storage time and both TPC (r = −0.974, *p* < 0.01) and ABTS (r = −0.931, *p* < 0.01), confirming progressive antioxidant degradation during storage. This trend is further supported by the moderate attenuation of DPPH activity (r = −0.491, *p* < 0.01) over the same interval. The near-linear inverse relationship between TPC and time indicates that phenolic degradation leads to antioxidant decline. Therefore, TPC serves as an indicator of antioxidant stability. This correlation indicates that the phenolic compounds are the contributors to the overall antioxidant activity in this matrix, aligning with findings in other plant-based materials where total phenolic content strongly correlates with antioxidant assays like ABTS and DPPH [39,40]. The degradation of antioxidant properties during storage was modeled using linear regression in SPSS (version 17.0, USA) to describe the relationship between total phenolic content (TPC) and time. Both zero-order and first-order kinetic models were evaluated, and the results are presented in Table 7.

The zero-order model described TPC degradation better than the first-order model, as indicated by its higher adjusted R^2^ values (0.931–0.971 vs. 0.888–0.953). This suggests that the rate of phenolic loss remained relatively constant throughout storage. The zero-order rate constants ranged from 30.796 to 35.101 mg GAE/100 g db per month across the four storage temperatures, while the first-order rate constants ranged from 0.090 to 0.103 month^−1^. Neither model showed a consistent trend with storage temperature, indicating that the small differences among treatments were within experimental variation rather than reflecting a temperature effect.

The predicted half-lives (L_50_) derived from the zero-order model were 210, 199, 207 and 220 days at 4, 25, 35 and 45 °C respectively, with corresponding first-order estimates of 222, 205, 213 and 234 days. Comparable zero-order degradation of phenolic content and antioxidant activity has been reported for other phenolic-rich plant extracts stored under low-moisture conditions: mango powder stored at 20–55 °C gave predicted shelf lives of 85–551 days depending on storage conditions [41], while facheiro fruit pulp powder with added polysaccharides showed a phenolic half-life of 85 days under a similar modelling approach [42]. The relatively slow decline in phenolic compounds in BLEP may be attributed to encapsulation within the maltodextrin matrix, which limits oxygen exposure to the particle surface, along with the low residual moisture content of the powder.

## 4. Conclusions

This study developed and optimized black lychee extracted powder (BLEP) using spray drying and evaluated its physicochemical characteristics and storage stability. Graphical optimization identified 17.80% maltodextrin and an inlet temperature of 200 °C as the optimum processing conditions within the experimental range. Maltodextrin concentration played a key role in determining powder quality by influencing moisture content, color stability, powder recovery, and the retention of phenolic compounds and antioxidant activity. The optimized BLEP contained vanillic acid and epicatechin as the predominant phenolic compounds, while leucine was the most abundant free amino acid.

During six months of storage at 4, 25, 35, and 45 °C, the optimized powder maintained good physicochemical stability, including low moisture content, stable color characteristics, and a consistent degree of glycation, whereas total phenolic content and antioxidant activity declined with storage time, with the ABTS assay showing the most pronounced decline over storage. These results indicate that phenolic degradation was the main contributor to the reduction in chemical antioxidant capacity during storage. The degree of glycation and HMF remained relatively stable. Overall, the optimized BLEP demonstrates potential as a shelf-stable bioactive food ingredient for dry food applications, particularly powdered formulations. Further studies are needed to investigate its bioaccessibility, in vitro digestion, sensory properties, and biological activities before commercial application.

## Figures and Tables

**Table 1:** Experimental runs obtained by varying maltodextrin concentration and inlet temperature.

Experiment	Maltodextrin Concentration (%)	Inlet Temperature (°C)
1	5.0	160
2	30.0	160
3	5.0	200
4	30.0	200
5	17.5	180
6	17.5	180

**Table 2:** Color characteristics, moisture content, and powder yield of BLEP with varying maltodextrin content and inlet temperature.

Physical Quality	Experiments					
	1	2	3	4	5	6
Color values						
- Lightness (L*)	62.17 ± 0.68	75.18 ± 0.75	59.26 ± 1.19	75.61 ± 0.36	72.99 ± 0.73	72.03 ± 0.75
- Redness (a*)	12.26 ± 0.04	8.97 ± 0.12	12.30 ± 0.14	8.87 ± 0.09	9.50 ± 0.09	9.49 ± 0.19
- Yellowness (b*)	22.02 ± 0.05	18.74 ± 0.19	20.42 ± 0.74	18.54 ± 0.19	19.74 ± 0.47	19.49 ± 0.75
- Chroma (C*)	26.46 ± 0.25	21.13 ± 0.41	24.88 ± 1.10	20.81 ± 0.15	21.72 ± 0.43	21.68 ± 0.43
- Hue angle (H°)	61.91 ± 0.27	64.67 ± 0.59	59.71 ± 0.91	64.76 ± 0.13	63.94 ± 0.35	64.42 ± 0.35
Moisture content (%)	5.08 ± 0.10	0.82 ± 0.09	4.48 ± 0.28	0.83 ± 0.35	0.09 ± 0.02	0.08 ± 0.10
Powder yield (%)	52.00	57.00	41.33	57.00	64.73	61.82

Data are presented as mean ± SD (*n* = 3). Yield was determined once per run.

**Table 3:** Chemical properties and antioxidant activities of BLEP produced under different maltodextrin concentrations and inlet temperatures.

Chemical Quality	Experiments					
	1	2	3	4	5	6
Total sugar content (g Glucose/100 g db)	59.13 ± 0.63	50.57 ± 0.53	59.18 ± 2.18	50.65 ± 1.14	46.82 ± 1.50	49.36 ± 1.49
Reducing sugar content (g Glucose/100 g db)	51.89 ± 3.28	30.28 ± 2.34	47.96 ± 1.51	29.03 ± 0.40	35.96 ± 1.70	34.42 ± 3.50
Total phenolic content (mg GAE/100 g db)	1203.52 ± 44.72	456.45 ± 10.26	1124.86 ± 25.39	396.02 ± 12.21	648.55 ± 20.52	581.83 ± 13.11
Total flavonoid content (mg QE/100 g db)	1544.14 ± 80.29	376.54 ± 37.78	1672.51 ± 152.18	344.12 ± 59.34	631.32 ± 64.68	582.75 ± 29.79
Antioxidant activity - DPPH (mg TE/100 g db)	691.67 ± 7.74	244.04 ± 13.11	659.97 ± 7.35	230.40 ± 5.78	373.83 ± 2.38	334.06 ± 7.10
- ABTS (mg TE/100 g db)	1560.52 ± 38.93	671.83 ± 13.29	1451.10 ± 88.22	674.13 ± 7.67	977.25 ± 7.67	952.82 ± 34.52
- FRAP (mg FeSO_4_/100 g db)	2215.19 ± 73.34	842.80 ± 34.57	2065.35 ± 137.14	707.78 ± 23.91	1182.10 ± 46.29	1047.12 ± 45.73

Data are presented as mean ± SD (*n* = 3); db = dry-weight basis.

**Table 4:** Individual sugar composition, rare sugars (allulose), and phenolic profiles of BLEP produced under different maltodextrin concentrations and inlet temperatures.

Chemical Quality	Experiments					
	1	2	3	4	5	6
Sugars (g/100 g db)						
- Allulose	0.39 ± 0.01	0.18 ± 0.02	0.50 ± 0.06	0.17 ± 0.04	0.29 ± 0.03	0.37 ± 0.01
- Fructose	6.36 ± 0.95	2.05 ± 0.46	5.33 ± 0.26	1.70 ± 0.24	2.60 ± 0.23	3.01 ± 0.45
- Mannose	0.87 ± 0.16	0.27 ± 0.09	0.79 ± 0.02	0.24 ± 0.03	0.36 ± 0.04	0.40 ± 0.05
- Glucose	12.62 ± 2.04	4.10 ± 0.91	10.21 ± 0.48	3.52 ± 0.44	5.00 ± 0.48	5.85 ± 0.83
Phenolics (mg/100 g db)						
- Protocatechuic acid	5.87 ± 0.12	2.47 ± 0.08	8.17 ± 0.06	2.78 ± 0.06	4.26 ± 0.09	3.95 ± 0.10
- Catechin	156.60 ± 0.81	38.69 ± 0.16	154.34 ± 0.55	52.64 ± 0.48	97.79 ± 0.12	89.70 ± 0.03
- Caffeine	109.03 ± 0.35	29.05 ± 0.12	103.86 ± 0.35	33.09 ± 0.58	55.87 ± 0.21	49.95 ± 0.09
- Vanillic acid	358.74 ± 1.25	197.44 ± 2.44	362.99 ± 1.14	203.86 ± 0.94	272.48 ± 0.08	252.72 ± 1.32
- Epicatechin	180.01 ± 2.02	47.65 ± 0.63	167.55 ± 0.63	57.02 ± 0.12	80.03 ± 0.94	81.50 ± 0.92
- Rutin	10.82 ± 0.38	ND	9.09 ± 0.19	ND	4.44 ± 0.05	4.34 ± 0.14
- *trans*-cinnamic acid	44.70 ± 0.44	2.98 ± 0.01	37.17 ± 0.80	2.52 ± 0.05	8.15 ± 0.20	6.95 ± 0.37

Data are presented as mean ± SD (*n* = 3); ND = not detected; db = dry-weight basis.

**Table 5:** Amino acid composition of BLEP produced under different maltodextrin concentrations and inlet temperatures.

Amino Profile (mg/100 g db)	Experiments					
	1	2	3	4	5	6
- Aspartic acid	4.58 ± 0.32	1.62 ± 0.01	4.74 ± 0.17	1.54 ± 0.15	2.41 ± 0.17	2.51 ± 0.10
- Threonine	ND	ND	ND	ND	ND	ND
- Serine	1.10 ± 0.05	0.48 ± 0.13	1.04 ± 0.06	0.36 ± 0.02	0.58 ± 0.02	0.48 ± 0.02
- Glutamic acid	5.34 ± 0.13	1.48 ± 0.12	4.83 ± 0.39	1.55 ± 0.23	2.41 ± 0.20	2.27 ± 0.01
- Proline	0.49 ± 0.01	0.16 ± 0.02	0.39 ± 0.01	0.16 ± 0.01	0.20 ± 0.01	0.20 ± 0.02
- Glycine	0.34 ± 0.01	0.12 ± 0.01	0.28 ± 0.01	0.12 ± 0.01	0.16 ± 0.01	0.16 ± 0.01
- Alanine	14.46 ± 0.10	4.67 ± 0.06	11.58 ± 0.01	4.62 ± 0.38	6.90 ± 0.03	7.11 ± 0.35
- Cysteine	ND	ND	ND	ND	ND	ND
- Valine	7.21 ± 0.42	1.50 ± 0.08	5.27 ± 0.09	1.52 ± 0.17	2.29 ± 0.23	2.12 ± 0.01
- Methionine	7.35 ± 0.18	0.28 ± 0.03	3.26 ± 0.23	0.59 ± 0.44	0.32 ± 0.12	0.42 ± 0.08
- Isoleucine	ND	ND	ND	ND	ND	ND
- Leucine	83.35 ± 1.13	26.78 ± 0.24	85.42 ± 1.31	30.43 ± 3.11	41.11 ± 0.20	41.01 ± 0.88
- Tyrosine	ND	ND	ND	ND	ND	ND
- Phenylalanine	1.73 ± 0.12	ND	0.96 ± 0.11	ND	ND	ND
- Histidine	ND	ND	ND	ND	ND	ND
- Lysine	1.05 ± 0.07	0.69 ± 0.02	0.90 ± 0.05	0.85 ± 0.01	0.87 ± 0.01	1.00 ± 0.01
- Arginine	1.23 ± 0.08	3.93 ± 0.25	4.34 ± 0.84	8.39 ± 0.43	7.09 ± 0.03	7.49 ± 0.19
Total amino acids	127.89 ± 2.61	41.58 ± 0.95	122.72 ± 3.26	50.01 ± 4.95	64.18 ± 2.22	64.61 ± 1.37

Data are presented as mean ± SD (*n* = 3); ND = not detected; db = dry-weight basis.

**Table 6:** Regression equations and associated model statistics for quality attributes of BLEP as affected by maltodextrin concentration and inlet temperature.

Quality Parameter	Regression Equation	*p*-Value	Adjusted R^2^
L* (lightness)	= 64.84 + 0.58(MD) − 0.03(T)	0.0417	0.7995
b* (Yellowness)	= 30.22 − 0.36(MD) − 0.05(T) + 0.01(MD × T)	0.0130	0.9783
Reducing sugar	= 64.09 − 0.81(MD) − 0.06(T)	0.0183	0.8841
Allulose	= 0.27 − 0.01(MD) + 0.01(T)	0.0164	0.8924
Fructose	= 9.26 − 0.16(MD) − 0.02(T)	0.0283	0.8453
Mannose	= 1.13 − 0.02(MD) − 0.01(T)	0.0309	0.8358
Glucose	= 18.65 − 0.30(MD) − 0.04(T)	0.0376	0.8129
DPPH	= 831.38 − 17.54(MD) − 0.57(T)	0.0192	0.8805
ABTS	= 1871.95 − 33.31(MD) − 1.34(T)	0.0061	0.9444
FRAP	= 2939.79 − 54.60(MD) − 3.56(T)	0.0231	0.8649
TFC	= 1516.28 − 49.92(MD) + 1.20(T)	0.0377	0.8127
TPC	= 1564.72 − 29.52(MD) − 1.74(T)	0.0212	0.8725
Protocatechuic acid	= 1.78 − 0.18(MD) + 0.03(T)	0.0209	0.8734
Catechin	= 148.86 − 4.39(MD) + 0.15(T)	0.0015	0.9782
Caffeine	= 118.78− 3.01(MD) − 0.01(T)	0.0153	0.8971
Vanillic acid	= 362.85 − 6.41(MD) + 0.13(T)	0.0038	0.9597
Rutin	= 15.63 − 0.40(MD) − 0.02(T)	0.0013	0.9800
Aspartic acid	= 4.89 − 0.12(MD) + 0.01(T)	0.0143	0.9017
Serine	= 1.54 − 0.03(MD) − 0.01(T)	0.0487	0.7778
Glutamic acid	= 6.49 − 0.14(MD) − 0.01(T)	0.0289	0.8432
Alanine	= 20.65 − 0.34(MD) − 0.04(T)	0.0236	0.8630

**Note:** MD = maltodextrin concentration (%); T = inlet temperature (°C).

**Table 7:** Zero-order and first-order kinetic parameters, adjusted R^2^ values, and predicted half-lives (L_50_) for total phenolic content of optimized BLEP during six months of storage at 4, 25, 35, and 45 °C.

**Temperature** **(°C)**	**Zero-Order**		**First-Order**	
	**Equation**	**Adjusted R^2^**	**Equation**	**Adjusted R^2^**
4	TPC = 446.928 − 32.407t	0.956	ln (TPC) = 6.122− 0.095t	0.953
25	TPC = 459.959 − 35.101t	0.943	ln (TPC) = 6.159 − 0.103t	0.925
35	TPC = 444.244 − 32.702t	0.931	ln (TPC) = 6.124 − 0.099t	0.888
45	TPC = 444.805 − 30.796t	0.971	ln (TPC) = 6.118 − 0.090t	0.951
**Temperature** **(** **°C** **)**	**k_0_** **(mg GAE/100 g db·Month^−1^)**	**L_50_ Zero-Order** **(Day)**	**k_1_** **(Month^−1^)**	**t½ First-Order** **(Day)**
4	32.407	210	0.095	222
25	35.101	199	0.103	205
35	32.702	207	0.099	213
45	30.796	220	0.090	234

t represents storage time (months). TPC expressed as mg GAE/100 g db. L_50_ calculated as C_0_/(2k_0_) for the zero-order model and as ln2/k_1_ for the first-order model. Day = 30.44 days per month.

## Data Availability

The original contributions presented in the study are included in the article/Supplementary materials; further inquiries can be directed to the corresponding author.

## Supplementary Materials

The following supporting information can be downloaded at: https://www.mdpi.com/article/10.3390/foods15162883/s1, Table S1: Color attributes and moisture content of BLEP during six months of storage under different temperature conditions; Table S2: Total phenolic content (TPC) and antioxidant activities of BLEP during six months of storage under different temperature conditions; Table S3: Changes in total sugar, reducing sugar, and degree of glycation (DG) of BLEP during storage for six months at different temperatures.
