# Supplementary material for: Optimization of Spray-Drying Conditions and Storage Stability of Black Lychee Extracted Powder: Impact of Maillard Conjugation on Bioactive Compounds and Antioxidant Activity
Source: Foods. 2026 Aug 18;15(16):2883. doi: 10.3390/foods15162883 (PMC13512327; doi:10.3390/foods15162883)
Supplement: Supplementary file 1 [file foods-15-02883-s001.zip › foods-4435467-supplementary.pdf]

Supplementary material file

Optimization of Spray Drying Conditions and Storage Stability of Black Lychee Extracted Powder: Impact of Maillard Conjugation on Bioactive Compounds and Antioxidant Activity

Supakit Chaipoot [1,2], Rewat Phongphisutthinant [1,2], Kuntathee Chaimueng [1], Sirinthip Jaijoi [1,5], Pairote Wiriyacharee[3], Chalermkwan Somjai [3], Worachai Wongwatcharayothin [4], Pattavara Pathomrungsiyounggul [5] and Kanokwan Kulprachakarn [6,*]

[1] Multidisciplinary Research Institute, Chiang Mai University, Chiang Mai 50200, Thailand
[2] Center of Excellence in Microbial Diversity and Sustainable Utilization, Chiang Mai University, Chiang Mai 50200, Thailand
[3] Processing and Product Development Factory, The Royal Project Foundation, Chiang Mai 50100, Thailand
[4] Faculty of Humanity, Chiang Mai University, Chiang Mai 50200, Thailand
[5] Faculty of Agro-Industry, Chiang Mai University, Chiang Mai 50100, Thailand
[6] School of Health Sciences Research, Research Institute for Health Sciences, Chiang Mai University, Chiang Mai 50200, Thailand
* Corresponding author E-mail: kanokwan.kul@cmu.ac.th

Table S1. Color attributes and moisture content of BLEP during six months of storage under different temperature conditions.

Time (month)	Temperature (°C)	Color values					Moisture content (%)
		L*	a*	b*	C*	H°	
0 (Control)	Room temp.	76.65±0.47	8.95±0.05	20.48±0.49	22.35±0.48	66.41±0.36	1.13±0.13
1	4	75.77±0.77	9.14±0.22	21.02±0.51	22.92±0.56	66.51±0.01	0.96±0.06
	25	75.62±0.09	9.07±0.11	21.52±0.06	23.25±0.09	66.92±0.09	0.75±0.35
	35	75.42±0.12	9.17±0.05	21.44±0.17	23.48±0.01	67.00±0.10	1.04±0.27
	45	76.25±0.15	9.06±0.05	21.29±0.06	23.14±0.08	66.94±0.06	1.07±0.27
2	4	75.64±0.03	8.97±0.04	21.29±0.04	23.10±0.04	67.10±0.03	0.77±0.05
	25	75.54±0.12	9.04±0.01	21.56±0.20	23.37±0.18	67.25±0.21	0.64±0.18
	35	76.21±0.02	8.84±0.09	21.06±0.20	22.84±0.22	67.23±0.02	0.63±0.01
	45	76.14±0.45	9.03±0.12	21.49±0.37	23.32±0.39	67.21±0.07	0.34±0.09
3	4	75.62±0.60	9.18±0.16	21.67±0.43	23.53±0.46	67.01±0.02	0.54±0.10
	25	75.90±0.25	8.95±0.04	21.34±0.04	23.14±0.06	67.26±0.06	0.55±0.12
	35	75.07±0.58	9.26±0.11	22.01±0.32	23.87±0.32	67.27±0.14	0.72±0.02
	45	75.51±0.41	9.45±0.18	21.68±0.22	23.61±0.23	66.67±0.04	0.52±0.08
4	4	75.63±0.24	9.06±0.04	21.43±0.11	23.26±0.07	67.08±0.21	0.27±0.05
	25	75.61±0.07	9.06±0.09	21.32±0.03	23.16±0.06	67.23±0.20	0.28±0.05
	35	76.21±0.06	9.18±0.02	21.32±0.07	23.22±0.07	66.71±0.04	0.36±0.01
	45	76.50±0.62	9.36±0.09	21.39±0.31	23.34±0.32	66.37±0.09	0.26±0.05
5	4	76.43±0.16	9.20±0.14	21.61±0.48	23.49±0.50	66.91±0.11	0.01±0.01
	25	76.26±0.17	9.02±0.09	21.27±0.28	23.10±0.29	67.01±0.05	0.03±0.01
	35	76.27±0.47	9.13±0.04	21.43±0.31	23.30±0.30	66.91±0.21	0.04±0.01
	45	75.76±0.19	9.44±0.01	21.78±0.12	23.73±0.11	66.56±0.09	0.03±0.01
6	4	75.73±0.29	9.19±0.04	21.49±0.04	23.37±0.04	66.85±0.04	0.01±0.01
	25	75.35±0.18	9.08±0.05	21.66±0.13	23.49±0.14	67.24±0.03	0.03±0.01

Time (month)	Temperature (°C)	Color values					Moisture content (%)
		L*	a*	b*	C*	H°	
	35	75.34±0.40	9.34±0.02	22.11±0.05	24.00±0.05	67.10±0.01	0.04±0.01
	45	75.42±0.29	9.58±0.09	22.13±0.38	24.12±0.38	66.53±0.16	0.03±0.01

Data are presented as mean ± SD (n = 3); Control = sample (month 0) stored at room temperature; No significance testing was conducted.

Table S2. Total phenolic content (TPC) and antioxidant activities of BLEP during six months of storage under different temperature conditions.

Time (month)	Temperature (°C)	TPC (mg GAE/100g db)	Antioxidant activity		
			FRAP (mg $FeSO_4$/100g db)	DPPH (mg TE/100g db)	ABTS (mg TE/100g db)
0 (Control)	Room temp.	437.11±8.72	862.06±42.22	305.71±16.36	555.98±62.98
1	4	424.17±7.78	903.43±54.30	197.38±13.05	418.41±27.46
	25	418.83±27.91	890.26±16.78	203.04±5.80	415.64±9.56
	35	416.44±11.49	876.57±17.11	208.32±9.56	431.31±9.59
	45	434.56±13.93	874.07±22.89	237.31±9.01	468.73±12.00
2	4	355.02±18.54	726.99±29.40	202.22±12.19	306.01±16.89
	25	356.98±14.43	789.32±64.12	203.13±4.61	307.94±17.96
	35	341.12±13.87	707.10±41.04	207.15±3.02	287.14±18.13
	45	336.43±9.82	711.54±31.76	217.18±6.33	343.09±31.77
3	4	330.55±7.38	772.87±9.77	214.23±7.12	97.60±10.04
	25	339.95±8.30	765.22±15.00	205.43±5.11	96.45±3.36
	35	336.89±9.90	776.99±15.26	220.53±3.25	103.79±5.98
	45	343.55±6.52	795.51±16.95	232.57±6.79	108.77±6.19
4	4	310.08±7.01	814.81±30.78	171.82±2.88	41.65±2.78
	25	318.97±7.90	796.67±19.12	171.70±7.57	36.78±2.28
	35	316.08±9.42	819.90±28.22	194.64±8.27	45.86±4.22
	45	322.47±6.23	872.50±48.17	206.12±6.28	53.74±2.71
5	4	295.88±11.41	796.47±13.76	157.43±28.81	54.30±6.50
	25	299.14±10.32	797.53±22.98	151.23±28.93	57.79±3.88
	35	304.99±11.84	795.35±30.80	186.41±26.20	57.39±4.38
	45	300.83±8.81	854.73±32.67	141.04±24.62	59.20±2.15
6	4	250.79±10.72	892.02±11.25	194.28±7.25	39.04±4.02
	25	235.36±7.88	879.75±12.44	200.37±6.21	38.58±1.34
	35	225.16±14.78	854.69±23.51	208.49±6.40	41.51±2.39
	45	252.31±17.44	932.09±12.44	203.26±6.47	43.51±2.48

Data are presented as mean ± SD (n = 3); db = dry weight basis. No significance testing was conducted.

Table S3. Changes in total sugar, reducing sugar, and degree of glycation (DG) of BLEP during storage for six months at different temperatures.

Time (month)	Temperature (°C)	Total sugar (g Glucose/100g db)	Reducing sugar (g Glucose/100g db)	DG (%)	HMF (g/kg)
0 (Control)	Room temp.	84.55±1.98	35.36±3.42	–	3.86±0.03
1	4	85.00±7.58	44.07±1.55	20.44±1.13	4.25±0.01
	25	93.53±1.47	44.00±1.68	21.55±1.81	3.88±0.05
	35	93.24±4.40	46.19±0.82	17.93±1.15	3.79±0.05
	45	83.69±0.75	41.03±3.19	18.81±2.18	3.91±0.01
2	4	79.02±4.11	43.82±1.56	16.63±1.9	3.70±0.02
	25	84.12±1.21	43.74±1.69	20.88±0.14	3.75±0.03
	35	84.42±1.31	45.95±0.83	22.96±0.93	3.85±0.02
	45	86.11±0.04	40.75±3.20	20.38±0.07	3.89±0.02
3	4	95.76±2.94	44.85±1.05	19.53±1.79	3.72±0.01
	25	94.19±2.40	45.95±0.17	18.67±2.65	3.75±0.03
	35	94.19±0.98	45.29±0.28	17.45±0.7	3.52±0.01
	45	96.56±2.98	43.58±1.15	16.62±6.81	3.40±0.08
4	4	98.94±0.97	45.44±1.08	18.59±0.14	3.74±0.06
	25	96.40±0.42	42.83±0.42	18.83±0.05	3.87±0.01
	35	96.29±0.84	43.41±1.00	16.59±3.01	3.74±0.03
	45	93.71±4.10	45.37±0.01	13.56±0.39	3.68±0.03
5	4	94.46±0.89	42.45±1.87	22.82±1.49	3.84±0.07
	25	93.87±2.34	40.04±0.43	20.93±2.36	3.97±0.02
	35	94.96±3.36	45.45±2.41	20.89±0.33	3.70±0.06
	45	96.14±0.0	41.71±0.84	18.84±3.61	3.74±0.05
6	4	97.38±1.61	40.39±1.81	20.13±1.51	3.69±0.01
	25	99.07±0.06	46.39±0.73	22.54±1.10	3.78±0.02
	35	94.90±0.65	52.05±0.01	21.67±0.22	3.64±0.06
	45	96.55±2.46	47.46±0.20	18.66±1.17	3.76±0.08

Data are presented as mean ± SD (n = 3); Control = sample (month 0) stored at room temperature. No significance testing was conducted.
